# A Real-World Study on the Quality of Life of Consumers with Dentine Hypersensitivity and the Benefits of Hypersensitivity Toothpaste Use

**DOI:** 10.3390/ijerph22020175

**Published:** 2025-01-27

**Authors:** Fatimah Maria Tadjoedin, Yuniarti Soeroso, Adityo Widaryono, Natalina Haerani, Yun Yee Amber Lee, Charles R. Parkinson, Sheryl S. L. Tan, Aida Gadzhieva-Moore, Rachel Lee-Yin Tan, Vandana Garg

**Affiliations:** 1 Department of Periodontology, Faculty of Dentistry, Universitas Indonesia, Salemba Raya No 4, Jakarta Pusat 10430, Indonesia; fatimah.tadjoedin@ui.ac.id (F.M.T.);; 2 Haleon, Lot 89, Jalan Enggang, Ampang/Hulu Kelang Industrial Estate, Ampang 68000, Selangor Darul Ehsan, Malaysia; 3 Haleon, St George’s Avenue, Weybridge KT13 0DE, UK; 4 Haleon, 23 Rochester Park, Singapore 139234, Singapore; 5 IQVIA Solutions Asia Pte Ltd., 79 Anson Road, Singapore 079906, Singapore

**Keywords:** quality of life, dentine hypersensitivity, hypersensitivity toothpaste, dental behavior, real-world evidence

## Abstract

Dentine hypersensitivity (DH) causes pain from exposed dentine, triggered by external stimuli. Limited evidence exists on its impact on quality of life (QoL), the effectiveness of hypersensitivity toothpaste (HT) and the dental behaviors of DH sufferers. This study therefore aimed to generate evidence to address these evidence gaps and validate the existing science behind the effectiveness of HT. An observational retrospective cross-sectional survey was conducted in Indonesia, which included the Dentine Hypersensitivity Experience Questionnaire-15 (DHEQ-15), items related to the perceived effectiveness of HT and self-reported dental health behaviors. A total of 511 respondents completed the survey. The mean (standard deviation) DHEQ score of participants was 82.44 (14.41), showing the substantial impact of DH on QoL. Nearly all HT users (97.7%) reported relief from DH, with additional benefits like long-lasting fresh breath (96.4%) and cleaner teeth (97.1%), with regular HT users experiencing greater benefits such as improvements in DH (R-HT users [4.65 (0.55)] vs. E-HT users [4.46 (0.65)], *p* < 0.01). Compared to HT users, mainstream toothpaste (MT) users were significantly less likely to brush their teeth at least three times a day (HT users [41.9%], MT users [18.7%], *p* < 0.05) and to have had a dental check-up within the past year (HT users [82.5%], MT users [47.3%], *p* < 0.05). This study found that DH significantly impacts QoL, and that HT effectively relieves DH symptoms, particularly for regular users, who also show better dental health behaviors than users of mainstream toothpaste. The preliminary results indicate that educating users about dental products, like hypersensitivity toothpaste, can improve oral health behaviors and outcomes.

## 1. Introduction

Dentine hypersensitivity (DH) is characterized by short, sharp pain arising in response to stimuli that are typically thermal, evaporative, tactile, osmotic or chemical and which cannot be ascribed to any other form of dental defect or disease [1]. This condition arises from exposure of dentine, the sub-surface layer beneath the enamel and cementum, which occurs due to various factors, including gingival recession, enamel erosion, periodontal treatment, improper brushing habits or a combination thereof [2]. To experience DH, the dentine of the tooth must be exposed, with dentine tubules patent to the oral environment (i.e., open at the exposed surface of the dentin through to vital dental pulp). Disruption of fluid in the tubules is thought to stimulate mechanoreceptors associated with odontoblast processes and nerve fibers located near the cervical pulp–dentine complex, triggering the short, sharp pain characteristic of dentine hypersensitivity [3].

DH is a widespread issue globally [4,5,6], affecting approximately one-third of adults at some point in their lives [7,8,9]. Beyond mere pain or discomfort, previous studies have shown that DH exerts a multifaceted impact encompassing significant impediments to a DH sufferer’s functional status and overall quality of life (QoL) [10,11]. Despite this evidence, there is little reference to the impact of DH on Indonesian’s QoL measured according to an oral condition-specific oral health quality of life (OHrQoL) measure. The Dentine Hypersensitivity Experience Questionnaire (DHEQ), comprising 48 items, is the first OHrQoL questionnaire developed to measure the impact of DH on an individual’s QoL from the perspective of the sufferer. The DHEQ aimed to measure particular everyday impacts related to DH. The questionnaire was developed through meticulously following a series of stages and adopting a robust theoretical framework. The validation of the DHEQ supports the feasibility of condition-specific instruments for measuring the biopsychosocial impacts of other oral conditions [12,13]. Subsequently, the DHEQ-15 was created as a concise version of the original DHEQ, designed to capture equivalent information with greater brevity [14]. Since its development, the measurement performance of the DHEQ-15, including internal consistency, test–retest reliability, and construct validity, has been rigorously validated around the world [15,16,17]. The DHEQ findings showed that the pain or discomfort associated with DH can result in the restriction of dietary choices and disruption of daily activities [18], as sufferers often modify their eating habits and lifestyle to mitigate their sensitivity [10]. Despite this understanding, there is currently a lack of data and no real-world study on how DH impacts individuals in Indonesia, a country known for its diverse and culturally ingrained culinary traditions and where social gatherings often revolve around food [19,20].

Desensitizing agents in hypersensitivity toothpastes (HTs), such as Novamin (a calcium sodium phosphosilicate bioactive glass), Stannous Fluoride, and Potassium Nitrate, have been shown in clinical trials to significantly reduce DH [21,22]. These desensitizing agents work via two primary mechanisms. Firstly, they may reduce fluid flow through the dentine by mechanically blocking or occluding the exposed dentine tubules, as in the case of Novamin (calcium sodium phosphosilicate) and Stannous Fluoride. Alternatively, these agents can decrease the activity of dentinal nerves, thereby inhibiting the transmission of pain signals to the central nervous system, as in the case of potassium ions [23]. Studies have shown that using HT twice daily on a regular basis ensures long-term benefits, including lasting sensitivity relief [24]. When formulated with fluoride, HT provides effective cavity protection. Further attributes such as fresh breath and whitening can be achieved by a judicious choice of toothpaste excipients [25]. However, there is little evidence of the real-world effectiveness of HT in the management of DH.

Therefore, the primary aims of this study were to assess the QoL of individuals suffering from DH using the DHEQ-15 and to evaluate the real-world effectiveness of the regular and continuous use of an HT. The secondary aim of this study was to gain insights into dental health behaviors (including patterns of HT use and attitudes and perceptions towards it).

## 2. Materials and Methods

### 2.1. Study Design

An observational retrospective cross-sectional survey study was conducted in Indonesia using a self-administered structured online questionnaire. The study protocol was developed collaboratively by experts from Haleon and IQVIA, aiming to generate evidence from adult Indonesian consumers with DH who used toothpaste. Recruitment and data collection took place from March to May 2024. Eligible participants were selected through an established online consumer panel, which sent email invitations to its members to participate in the survey. Interested individuals were directed to an informed consent form, where they confirmed their willingness to participate. After providing consent, participants underwent an eligibility screening process. Only those who met the inclusion criteria and agreed to participate were invited to complete the survey. All the recruited participants provided written informed consent prior to participation.

Ethical approval was obtained from the Ethical Commission for Dental Research (KEPKG), Faculty of Dentistry, Universitas Indonesia (approval reference number: 17/Ethical Approval/FKGUI/III/2024).

### 2.2. Online Survey

The online questionnaire included items on sociodemographics and the severity of DH (frequency and duration of DH), the DHEQ-15, items related to the effectiveness of HT, and items related to dental health behavior (including patterns of toothpaste usage and attitudes towards and perceptions of HT). The online questionnaire was first developed in English before being translated into Bahasa Indonesia. Translation accuracy was ensured by having a local team review and validate the translations. The full questionnaire can be found in Supplementary File S1.

The DHEQ-15 was selected for use in this study over the original DHEQ due to its brevity. The DHEQ-15 is a validated short-form version of the DHEQ, which originally comprised 48 items. The items included in the DHEQ-15 from the original DHEQ were determined by DH patients’ most frequently experienced items and those most important to them [14]. The fifteen items cover five domains of daily functional restrictions, coping behaviors, personal identity, social impact and emotional impact measured on a 7-point Likert scale (‘7—Strongly agree’, ‘6—Agree’, ‘5—Agree a little’, ‘4—Neither agree or disagree’, ‘3—Disagree a little’, ‘2—Disagree’, or ‘1—Strongly disagree’). The domain and total scores are obtained by summing the individual values. The total score ranges from a minimum of 15 to a maximum of 105, with higher scores indicating greater impairment to the respondents’ QoL.

### 2.3. Sample

Members of the adult population of Indonesia with DH were recruited from an established online consumer panel between March and May 2024. The panel sent email invitations to its members, inviting them to participate in the survey. Interested individuals were directed to an informed consent form, where they indicated their willingness to participate. Following this, consenting participants underwent an eligibility screening process. During the informed consent and screening process, participants were blinded to the specific product being assessed (i.e., Sensodyne toothpaste) to reduce bias during recruitment. The inclusion criteria were as follows: aged 18–65 years old, residing in Indonesia (including: Jakarta, Sumatera, Kalimantan, Sulawesi, Bali Nusra, Central Java, West Java or East Java), has experienced DH/sensitive teeth symptoms/or has been diagnosed with DH by a dentist, uses toothpaste in his/her daily dental hygiene routine, brushes his/her teeth twice or more daily, has used any toothpaste to alleviate DH in the past 3 months and has provided informed consent to participate in this study.

The following exclusion criteria were applied: have used partial dentures or full dentures in the past 3 months, have had severe periodontal disease diagnosed by a dentist in the past 3 months, have had active tooth decay that was causing pain in the past 3 months, have undergone dental surgeries (e.g., dental bone graft, periodontal surgery or jaw surgery) in the past 3 months, have experienced chronic conditions that require long-term pain medication (i.e., pain medication intake for more than 3 days a week) in the past 3 months, is an employee/contractor or immediate family member of the PI, Research Vendor for Recruitment, or Sponsor of this study or companies that produce toothpaste.

To determine the appropriate sample size for this study, the following formula was used: [(Z-score)^2^ × SD × (1 − SD)/(margin of error)^2^]. A margin of error of 5%, a standard deviation (SD) of 0.5 and a confidence level of 95% were considered. Under these parameters, a sample size of approximately 400 would be sufficient. However, an additional 100 participants were deemed necessary to ensure an adequate group representation and statistical power for analyzing the differences between three distinct groups defined by their toothpaste usage patterns. The first two groups consisted of respondents who used HT (any of the following: Sensodyne Deep Clean/Sensodyne 24/7 Protection Fresh Mint/Sensodyne Cool Gel/Sensodyne Repair & Protect/Sensodyne Herbal/Sensodyne Original/Sensodyne Multi-Action), with the first group being regular HT users (respondents who used HT to brush their teeth at least twice daily for at least 4 days a week, over the past 3 months), thereafter referred to as R-HT (regular HT) users, and the second group being episodic HT users (respondents who used HT to brush their teeth at least twice daily for less than 4 days a week/only used HT when suffering from hypersensitivity and stopped afterwards, over the past 3 months), thereafter referred to as E-HT (episodic HT) users. The last group consisted of users of mainstream toothpastes (respondents who used any of the following non-HTs: Pepsodent Pasta Gigi Berlubang/Pepsodent Complete 8 Herbal/Pepsodent Complete 8 Multi-Protection/Ciptadent Maxi Complete/Ciptadent Maxi Herbal/Close Up Everfresh) and who had not used HT in the past 3 months, thereafter referred to as MT (mainstream toothpaste) users. The final sample size required was minimally N = 500 (minimum R-HT users: n = 80, minimum E-HT users: n = 80, minimum MT users: n = 200).

Specific quotas were established to ensure a representative sample based on age and area of residence. A minimum of n = 200 participants aged between 26 and 50 years old was required. Additionally, a maximum of n = 200 participants was to be recruited from Central Java, West Java and East Java, with the remaining quota for participants from Jakarta, Sumatera, Kalimantan, Sulawesi and Bali Nusra.

### 2.4. Data Analysis

Only completed questionnaires were analyzed. Respondent characteristics and demographics were summarized using descriptive statistics. Continuous variables were reported as means and SDs within brackets, while categorical variables were presented as frequencies and proportions. For group comparisons, chi-square tests and Student’s *t*-test were employed to analyze categorical and continuous variables, respectively. Differences between groups were considered significant at the 5% level (*p* < 0.05). All data management and analyses were performed using Wincross V21 (data tabulation) and SPSS V23 (data cleaning and formatting).

## 3. Results

### 3.1. Demographic and Clinical Characteristics of Sample

A total of 511 respondents completed the survey, of which, 308 were HT users (226 R-HT users and 82 E-HT users) and 203 were MT users. The sociodemographic characteristics of the respondents are summarized in Table 1. The mean (SD) age of the sample was 39.3 (9.37) years. The majority of respondents were female (56.5%), of middle income (52.6%) and resided in Jakarta/Sumatera/Kalimantan/Sulawesi/Bali Nusra (60.5%). A significant difference in monthly household income and area lived in was observed between HT users and MT users. HT users reported a significantly higher mean (SD) income of IDR 5,556,818 (1,659,884) [USD 341.50 (102.00)], compared to IDR 5,059,360 (2,118,959) [USD 310.90 (130.21)], (*p* < 0.05) for MT users, (*p* < 0.01), and they were more likely to reside in Jakarta/Sumatera/Kalimantan/Sulawesi/Bali Nusra (HT users [73.7%], MT users [40.4%], *p* < 0.01).

The clinical characteristics of the respondents are summarized in Table 2. Almost all respondents indicated that all their teeth were natural (93.7%). Most respondents reported experiencing a moderate DH duration defined as lasting between 1 to 20 min (64.6%) and DH of high frequency defined as several times on a daily/weekly basis (61.6%). Most respondents reported experiencing at least 2 food/drink sensitivity symptoms (sensitivity to hot, cold, sweet or sour) (57.7%).

### 3.2. DH Impact on Quality-of-Life

**Figure 1:**
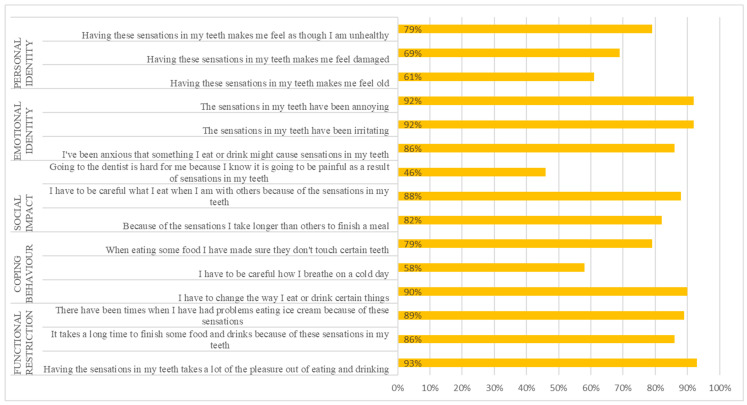
Proportion of respondents who were impacted by DH.

The mean DHEQ scores are presented in Table 3. The mean (SD) DHEQ score of all the respondents was 82.44 (14.41), suggesting that DH had a substantial negative impact on the respondents’ QoL. The mean (SD) DHEQ scores were significantly higher in respondents experiencing a higher frequency of DH compared to respondents experiencing a lower frequency of DH (high frequency: 87.23 [12.06], low frequency: 74.73 [14.56], *p* < 0.01). Similarly, the mean (SD) DHEQ scores were higher for respondents who reported experiencing a long duration for DH episodes compared to a short duration (short duration: 75.73 [15.30], long duration: 84.92 [11.17], *p* < 0.01). The same patterns were observed for the mean DHEQ domain scores. The biggest impact of DH perceived by respondents was in functional restriction and emotional identity, with 93.0%, 92.0% and 92.0% of respondents agreeing that ‘having sensations in my teeth takes a lot of the pleasure out of eating and drinking’, ‘the sensations in my teeth have been irritating’ and ‘the sensations in my teeth have been annoying’, respectively (Figure 1).

### 3.3. Effectiveness of Hypersensitivity Toothpaste in Management of DH

Table 4 provides details on the perceived effectiveness of HT as reported by its users. Nearly all HT users (97.7%) noted an improvement in their DH after using HT. Furthermore, 95.8% of HT users reported experiencing fewer DH episodes following the use of it. A total of 94.1% of HT users reported that since using HT, they were able to eat the food that they liked without worrying about tooth sensitivity. In addition to relief from DH, HT users also reported benefits such as a long-lasting feeling of fresh breath (96.4%) and cleaner teeth (97.1%). Among R-HT users, 96.9% reported fresh breath, 97.8% reported experiencing clean teeth, 97.4% reported being able to enjoy food without being bothered by tooth sensitivity and 96.0% reported feeling less pain when having hot and cold food and drinks.

Notably, R-HT than E-HT users indicated higher agreement in the improvement of DH (R-HT users [4.72 (0.49)] vs. E-HT users [4.46 (0.65)], *p* < 0.01), a reduction in DH episodes (R-HT users [4.65 (0.54)] vs. E-HT users [4.32 (0.80)], *p* < 0.01) and an increase in the enjoyment of their favorite food without sensitivity concerns (R-HT users [4.58 (0.59)] vs. E-HT [4.35 (0.69)], *p* < 0.01) (Table 5). Among HT users who have previously used other HT brands (n = 150), 96.7% reported that Sensodyne provided better relief from DH compared to the other HT brands they had used (Table 6).

Results concerning the dental health behavior of the respondents are presented in Table 7. The majority of respondents reported brushing their teeth twice daily (67.3%), having their last dental check-up within the past year (68.5%) and choosing to use sensitivity toothpaste to relieve their DH (76.3%). A significantly higher proportion of HT users had been formally diagnosed with DH by a dentist compared to MT users (HT [89.6%], MT users [32.0%], *p* < 0.01). Compared to HT users, MT users were significantly less likely to brush their teeth at least three times a day (HT users [41.9%], MT users [18.7%], *p* < 0.01). Additionally, MT users were significantly less likely than HT users to have had a dental check-up within the past year (HT users [82.5%], MT users [47.3%], *p* < 0.01). Notably, HT was reported as the most frequently used treatment for alleviating symptoms of DH, with three in four respondents (76.3%) indicating its use. Among E-HT users, 67.1% reported using HT only for short periods until their DH improved. Interestingly, a significantly higher proportion of MT users took painkillers compared to HT users to alleviate pain from DH (HT users [10.7%], MT users [34.5%], *p* < 0.01), and almost one in five MT users (18.7%) reported that they did not use any treatment for their DH.

### 3.4. Reasons for Using HT

Table 8 outlines the reasons for selecting HT to manage DH. Among HT users, the predominant reason for choosing to use HT was due to their belief that regular toothpaste was insufficient to address their DH (68.2%) (R-HT users [69.5%] and E-HT users [64.6%]). Slightly over half the HT users indicated that they chose to use HT because they understood how it effectively addresses DH (50.3%). Among R-HT users, the primary motivation indicated for continued use was the perceived effective relief from DH (86.3%). Other motivations for using HT regularly included feeling cleaner teeth (67.3%), experiencing healthier gums (69.0%) and stronger teeth (66.4%), having fresh breath (52.7%) and feeling protected from cavities (57.5%). Notably, 71.2% of R-HT users reported that they experienced multiple oral health benefits (healthier gums, cleaner teeth, stronger teeth, teeth protection and/or fresh breath) in addition to relief from DH.

### 3.5. Attitude and Perception Towards HT

Table 9 presents the results regarding attitudes and perceptions towards HT. Nearly all HT users reported enjoying the flavor of HT toothpaste (96.1%) and that they could use it every day (94.2%). In addition, 98.6% of R-HT users agreed that HT is the best choice for sensitive teeth. Almost all HT users (99.0%) trusted HT to help manage their DH, and 86.0% of HT users reported that they would definitely repurchase HT. A total of 95.1% of HT users would recommend HT to other consumers suffering from DH. A large majority (93.8%) of HT users also reported believing that the use of HT prevents DH from recurring.

Among R-HT users, 96.5% reported believing that the regular use of HT prevented their teeth sensitivity from worsening. Notably, 100.0% of R-HT users reported being satisfied with the product, and nearly all (98.6%) agreed that HT was the best choice for their sensitive teeth. Additionally, 92.0% of R-HT users believed that regular HT use is the best choice for their sensitive teeth, given its five-in-one oral health benefits in relation to cleaning, fresh breath, pain/sensitivity relief, DH protection (prevents recurrence) and DH protection (prevents symptoms worsening).

Notably, the level of satisfaction with HT (R-HT users [4.90 (0.38)], E-HT users [4.67 (0.50)], *p* < 0.01), trust in the effectiveness of HT (R-HT users [4.85 (0.38)], E-HT users [4.71 (0.51)], *p* < 0.01), the likelihood of continued purchase (R-HT users [4.88 (0.52)], E-HT users [4.59 (0.83)], *p* < 0.05), and recommendation intent to other consumers (R-HT users [4.74 (0.64)], E-HT users [4.26 (0.78)], *p* < 0.01) were significantly higher among regular R-HT users compared to E-HT users (Table 10).

## 4. Discussion

DH is highly prevalent across the world, with its rates varying slightly between different countries [5,6,8,9]. The high prevalence of DH around the world is reflected in the increasing demand for preventive oral healthcare products. Today, the treatment of DH is increasingly diversified and accessible to the public, to help improve oral health in general [26]. This study evaluated the impact of DH on QoL, assessed the real-world effectiveness of regular HT use and explored dental health behaviors by providing insights into patterns of HT usage, as well as attitudes and perceptions towards the product among consumers.

The DHEQ [12] and its abbreviated version, DHEQ-15 [14], are reliable and valid measures of the DH experience. They serve as a useful addition to the growing number of condition-specific OHrQoL instruments for facilitating our understanding of the biopsychosocial impacts of oral health conditions. Using the DHEQ-15, the results from this study indicate that DH has a substantial negative impact on QoL among DH sufferers, with pronounced impacts on both functional restriction and emotional identity. The results revealed that individuals with DH experience considerable limitations in their daily activities, particularly those involving eating and drinking, which can diminish their overall enjoyment and QoL. Additionally, the emotional impact of DH was found to be considerable. These results validated findings from previous studies that the impact of DH extends beyond physical pain or discomfort, significantly affecting both functional and emotional health [10,11,27], underscoring the need for effective management strategies to mitigate its impact.

The current available treatments for dentine DH include surgical interventions, laser treatment and HT use [28,29,30]. Among these options, HT is non-invasive and is the most economical solution. The results of this study support HT as an effective method for managing DH and providing its users with multiple oral health benefits, including clean teeth, long-lasting fresh breath, being able to enjoy food without being bothered by tooth sensitivity, prevention against recurring sensitivity and prevention of the condition worsening. The perceived effectiveness of HT was notably higher among R-HT users compared to E-HT users. Notably, although both R-HT and E-HT users reported experiencing the benefits from HT use, satisfaction, trust in the effectiveness of HT, the likelihood of continued purchases and recommendation intent were higher among R-HT users than among E-HT users. These findings highlight the importance of not only utilizing HT to manage DH but using it consistently to maximize its benefits, supporting the recommendation for the regular use of HT to achieve optimal and sustained relief from DH [31].

The findings from this study show that HT users exhibit better dental health behaviors compared to those who are users of MT. Specifically, HT users reported brushing their teeth more frequently, visiting the dentist within the past year and receiving a formal diagnosis of DH from a dentist. These behaviors may be attributed to better oral health literacy and education [32]. This is supported by the findings in this study, where HT users reported choosing sensitivity toothpaste as their preferred method for managing DH largely due to their understanding of the toothpaste’s mechanism of action. These findings highlight the critical role of oral health literacy in promoting better dental care practices [33] and the need for education to improve oral health, including the education of consumers on the science of toothpaste ingredients to make the right selection for their oral health conditions.

One of the key strengths of this study is that it generates data directly from consumers of HT. This consumer-centered approach provides valuable insights from the actual users of these products. Additionally, the study includes data on the perceived effectiveness of HT in real-world use, allowing us to capture real-world experiences and outcomes. This is particularly advantageous as clinical trials may not reflect everyday use, while real-world data can reveal how these technologies perform in diverse, less controlled environments. By presenting consumer-derived data, this study offers a more comprehensive understanding of HT’s effectiveness and applicability in everyday settings. We acknowledge that this study has several limitations. Firstly, the recruitment requirement of having access to a digital device to complete the online survey may have excluded individuals with limited access to technology. Secondly, the study collected self-reported data, which might be subject to recall bias. However, this is mitigated by recruiting consumers who used sensitivity toothpaste recently (within the past 3 months). Fourthly, this study exclusively recruited users of Sensodyne to represent the broader category of HT users. Consequently, the findings may not be generalizable to individuals using other brands of HT. Lastly, factors such as dietary habits linked to socio-economic status and access to dental care (especially in rural areas) can all influence oral health outcomes but may not be adequately accounted for in this study.

## 5. Conclusions

This study demonstrated that DH sufferers experience a poor QoL (their mean (SD) DHEQ score was 82.44 (14.41)), particularly experiencing functional restriction and problems with emotional identity. Importantly, this study also found that HT effectively alleviates DH, with 97.7% of HT users reporting improvement in their DH after using HT, with regular HT users experiencing greater relief, satisfaction and benefits compared to those who used the product episodically. The preliminary results suggest that educating users about dental products, like HT, can improve oral health behaviors and outcomes.

## Figures and Tables

**Table 1:** Sociodemographic characteristics of respondents with DH.

	Overall (N = 511)	Total HT * Users (N = 308)	R-HT * Users (N = 226)	E-HT * Users (N = 82)	MT ^#^ Users (N = 203)
Age, years					
Mean (SD)	39.3 (9.37)	39.8 (8.96)	38.2 (8.37)	44.2 (9.06)	38.7 (9.95)
					*p* = 0.195 ^+^
Sex, n (%)					
Male	222 (43.4)	130 (42.2)	93 (41.2)	37 (45.1)	92 (45.3)
Female	289 (56.6)	178 (57.8)	133 (58.8)	45 (54.9)	111 (54.7)
					*p* = 0.488 ^+^
Monthly household income, IDR					
Mean (SD)	5,359,198 (1,869,836)	5,556,818 (1,659,884)	5,396,018 (1,713,588)	6,000,000 **^+^** (1,419,507)	5,059,360 (2,118,959)
					*p* < 0.01 ^+^
Monthly household income, n (%)					
Low (less than IDR 2,700,000)	41 (8.0)	10 (3.2)	8 (3.5)	2 (2.4)	31 (15.3)
Middle (IDR 2,700,001 to 4,500,000)	247 (52.6)	162 (54.9)	124 (46.3)	38 (41.9)	85 (52.6)
High (IDR 4,500,001 and above)	223 (44.2)	136 (41.6)	94 (51.2)	42 (42.9)	87 (44.2)
Area lived in, n (%)					
Jakarta/Sumatera/Kalimantan/Sulawesi/Bali Nusra	309 (60.5)	227 (73.7)	174 (77.0)	53 (64.6)	82 (40.4)
Central Java/West Java/East Java	202 (39.5)	81 (26.3)	52 (23.0)	29 (35.4)	121 (59.6)
					*p* < 0.01 ^+^

Abbreviation: E-HT, episodic hypersensitivity toothpaste; DH, dentine hypersensitivity; HT, hypersensitivity toothpaste; IDR, Indonesian rupiah; MT, mainstream toothpaste; R-HT, regular hypersensitivity toothpaste; SD, standard deviation. * HT refers to Sensodyne Deep Clean/Sensodyne 24/7 Protection Fresh Mint/Sensodyne Cool Gel/Sensodyne Repair & Protect/Sensodyne Herbal/Sensodyne Original/Sensodyne Multi-Action. ^#^ MT refers to Pepsodent Pasta Gigi Berlubang/Pepsodent Complete 8 Herbal/Pepsodent Complete 8 Multi-Protection/Ciptadent Maxi Complete/Ciptadent Maxi Herbal/Close Up Everfresh. ^+^ *p* < 0.05, compared to total Sensodyne users by *t*-test for means, paired/overlap Z-test for percentages.

**Table 2:** Clinical characteristics of respondents with DH.

	Overall (N = 511)
Dental profile, n (%)	
All of my teeth are my own natural teeth	479 (93.7)
I have most of my natural teeth and a few dental crowns/bridges/implants	28 (5.5)
I use fixed or removable orthodontic braces/bands/fixed orthodontic retainer	9 (1.8)
Food/drink Sensitivity, n (%) ^a^	
To hot food/drinks	163 (31.9)
To cold food/drinks	460 (90.0)
To sweet food/drinks	168 (32.9)
To sour food/drinks	195 (38.2)
DH frequency, n (%)	
High (several times on a daily/weekly basis)	315 (61.6)
Low (several times on a monthly basis)	196 (38.4)
DH duration, n (%)	
Short (<1 min)	121 (23.8)
Moderate (1–20 min)	330 (64.6)
Long (>20 min)	60 (11.7)
Number of food/drink sensitivity symptoms, n (%)	
1 symptom	216 (42.3)
2 or more symptoms	295 (57.7)

Abbreviations: DH, dentine hypersensitivity. ^a^ As multiple options can be selected, the sum of proportions across all categories may exceed 100%.

**Table 3:** DHEQ scores of all respondents.

	DHEQ Total Score *	DHEQ Functional Restriction	DHEQ Coping Behavior	DHEQ Social Impact	DHEQ Emotional Identity	DHEQ Personal Identity
All respondents, mean (SD)	82.44 (14.41)	17.65 (2.98)	15.99 (3.52)	15.64 (3.24)	17.82 (2.82)	15.35 (4.30)
DH frequency, mean (SD)						
Low ^a^ (n = 196)	74.73 (14.56)	16.22 (3.31)	14.12 (3.51)	14.40 (3.24)	16.48 (3.21)	13.52 (4.24)
High ^b^ (n = 315)	87.23 (12.06)	18.54 (2.35)	17.15 (2.98)	16.41 (3.00)	18.64 (2.18)	16.49 (3.93)
	*p* < 0.01	*p* < 0.01	*p* < 0.01	*p* < 0.01	*p* < 0.01	*p* < 0.01
DH duration, mean (SD)						
Short (n = 121)	75.73 (15.30)	16.40 (3.56)	14.64 (3.77)	14.47 (3.50)	16.35 (3.34)	13.87 (4.08)
Moderate (n = 330)	84.45 (13.87)	17.94 (2.71)	16.42 (3.33)	16.25 (3.09)	18.12 (2.55)	15.72 (4.37)
Long (n = 60)	84.92 (11.17)	18.57 (2.27)	16.33 (3.34)	14.63 (2.55)	19.08 (2.18)	16.30 (3.65)
	*p* < 0.01 **^#^**	*p* < 0.01 **^#^**	*p* < 0.05 **^#^**	*p* = 0.724 **^#^**	*p* < 0.01 **^#^**	*p* < 0.01 **^#^**

Abbreviations: DH, dentine hypersensitivity; DHEQ, Dentine Hypersensitivity Experience Questionnaire; SD, standard deviation. * DHEQ total score ranges from a minimum of 15 to a maximum of 105, with higher scores indicating greater impairment in quality of life. ^a^ Experienced DH several times on a daily/weekly basis. ^b^ Experienced DH several times on a monthly basis. ^#^ Comparing groups with short and long DH durations, by *t*-test.

**Table 4:** Perceived effectiveness of Sensodyne.

	Total HT * Users (N = 308)	R-HT * Users (N = 226)	E-HT * Users (N = 82)
My dental hypersensitivity has improved after using Sensodyne, n (%)			
Completely disagree	0 (0.0)	0 (0.0)	0 (0.0)
Somewhat disagree	2 (0.6)	1 (0.4)	1 (1.2)
Neither agree nor disagree	5 (1.6)	1 (0.4)	4 (4.9)
Somewhat agree	91 (29.5)	58 (25.7)	33 (40.2)
Completely agree	210 (68.2)	166 (73.5)	44 (53.7)
			*p* < 0.05 ^#^
I experience less frequent dental hypersensitivity episodes after using Sensodyne, n (%)			
Completely disagree	1 (0.3)	0 (0.0)	1(1.2)
Somewhat disagree	3 (1.0)	1 (0.4)	2 (2.4)
Neither agree nor disagree	9 (2.9)	4 (1.8)	5 (6.1)
Somewhat agree	103 (33.4)	67 (29.6)	36 (43.9)
Completely agree	192 (62.3)	154 (68.1)	38 (46.3)
			*p* < 0.05 ^#^
Since using Sensodyne, I am able to eat the food that I like without worrying about tooth sensitivity, n (%)			
Completely disagree	0 (0.0)	0 (0.0)	0 (0.0)
Somewhat disagree	2 (0.6)	1 (0.4)	1 (1.2)
Neither agree nor disagree	16 (5.2)	9 (4.0)	7 (8.5)
Somewhat agree	111 (36.0)	75 (33.2)	36 (43.9)
Completely agree	179 (58.1)	141 (62.4)	38 (46.3)
			*p* = 0.13 ^#^
With regular use of Sensodyne/with Sensodyne, I can enjoy eating sweets and desserts without being bothered by tooth sensitivity, n (%)			
Completely disagree	0 (0.0)	0 (0.0)	0 (0.0)
Somewhat disagree	0 (0.0)	0 (0.0)	0 (0.0)
Neither agree nor disagree	11 (3.6)	6 (2.7)	5 (6.1)
Somewhat agree	121 (39.3)	82 (36.3)	39 (47.6)
Completely agree	176 (57.1)	138 (61.1)	38 (46.3)
Regular use of Sensodyne/Sensodyne provides me with a long-lasting feeling of fresh breath, n (%)			
Completely disagree	0 (0.0)	0 (0.0)	0 (0.0)
Somewhat disagree	2 (0.6)	1 (0.4)	1 (1.2)
Neither agree nor disagree	11 (3.6)	6 (2.7)	5 (6.1)
Somewhat agree	107 (34.7)	71 (31.4)	36 (43.9)
Completely agree	188 (61.1)	148 (65.5)	40 (48.8)
Regular use of Sensodyne/Sensodyne cleans my teeth well, n (%)			
Completely disagree	1 (0.3)	0 (0.0)	1 (1.2)
Somewhat disagree	0 (0.0)	0 (0.0)	0 (0.0)
Neither agree nor disagree	8 (2.6)	5 (2.2)	3 (3.7)
Somewhat agree	69 (22.4)	40 (17.7)	29 (35.4)
Completely agree	230 (74.7)	181 (80.1)	49 (59.8)
With regular use of Sensodyne/with Sensodyne, I feel less pain when I have hot and cold food and drinks, n (%)			
Completely disagree	1 (0.3)	1 (0.4)	0 (0.0)
Somewhat disagree	2 (0.6)	1 (0.4)	1 (1.2)
Neither agree nor disagree	11 (3.6)	7 (3.1)	5 (6.1)
Somewhat agree	105 (34.1)	66 (29.2)	39 (47.6)
Completely agree	188 (61.1)	151 (66.8)	37 (45.1)

Abbreviation: E-HT, episodic hypersensitivity toothpaste; HT, hypersensitivity toothpaste; R-HT, regular hypersensitivity toothpaste. * HT refers to Sensodyne Deep Clean/Sensodyne 24/7 Protection Fresh Mint/Sensodyne Cool Gel/Sensodyne Repair & Protect/Sensodyne Herbal/Sensodyne Original/Sensodyne Multi-Action. ^#^ Comparing E-HT users with R-HT users who selected ‘somewhat agree’/‘completely agree’, using paired/overlap Z-test for percentages.

**Table 5:** Mean (SD) score of perceived effectiveness of HT (Sensodyne).

	HT * Users (N = 308)	R-HT * Users (N = 226)	E-HT * Users (N = 82)
My dental hypersensitivity has improved after using Sensodyne	4.65 (0.55)	4.72 (0.49)	4.46 (0.65)
			*p* < 0.01 ^#^
Sensodyne provides better relief for my dental hypersensitivity as compared to other toothpaste brands I have used *	4.66 (0.57)	4.76 (0.46)	4.53 (0.66)
			*p* < 0.01^#^
I enjoy Sensodyne’s flavor	4.63 (0.60)	4.72 (0.51)	4.37 (0.76)
			*p* < 0.01 ^#^
I experience less frequent dental hypersensitivity episodes after using Sensodyne	4.56 (0.63)	4.65 (0.54)	4.32 (0.80)
			*p* < 0.01 ^#^
Since using Sensodyne, I am able to eat the food that I like without worrying about tooth sensitivity	4.52 (0.63)	4.58 (0.59)	4.35 (0.69)
			*p* < 0.01 ^#^
I can use Sensodyne every day	4.65 (0.65)	4.85 (0.37)	4.12 (0.91)
			*p* < 0.01 ^#^

Abbreviation: E-HT, episodic hypersensitivity toothpaste; HT, hypersensitivity toothpaste; R-HT, regular hypersensitivity toothpaste. * HT refers to Sensodyne Deep Clean/Sensodyne 24/7 Protection Fresh Mint/Sensodyne Cool Gel/Sensodyne Repair & Protect/Sensodyne Herbal/Sensodyne Original/Sensodyne Multi-Action. ^#^ Comparing E-HT users with R-HT users by *t*-test for means.

**Table 6:** Perceived effectiveness of Sensodyne relative to other toothpaste brands.

	Total HT * Users ^#^ (N = 150)	R-HT * Users ^#^ (N = 84)	E-HT * Users ^#^ (N = 66)
**Sensodyne provides better relief for my dental hypersensitivity as compared to other toothpaste brands I have used, n (%)**			
Completely disagree	0 (0.0)	0 (0.0)	0 (0.0)
Somewhat disagree	1 (0.7)	0 (0.0)	1 (1.5)
Neither agree nor disagree	4 (2.7)	1(1.2)	3 (4.5)
Somewhat agree	40 (26.7)	18 (21.4)	22 (33.3)
Completely agree	105 (70.0)	65 (77.4)	40 (60.6)

Abbreviation: E-HT, episodic hypersensitivity toothpaste; HT, hypersensitivity toothpaste; R-HT, regular hypersensitivity toothpaste. * HT refers to Sensodyne Deep Clean/Sensodyne 24/7 Protection Fresh Mint/Sensodyne Cool Gel/Sensodyne Repair & Protect/Sensodyne Herbal/Sensodyne Original/Sensodyne Multi-Action. ^#^ HT users who have tried other brands of HT3. 4. Dental health behavior.

**Table 7:** Dental health behavior of respondents with DH.

	Overall (N = 511)	HT * Users (N = 308)	R-HT * Users (N = 226)	E-HT * Users (N = 82)	MT ^#^ Users (N = 203)
Has been diagnosed with DH, n (%)					
Yes	341 (66.7)	276 (89.6)	210 (92.9)	66 (80.5)	65 (32.0)
No	170 (33.3)	32 (10.4)	16 (7.1)	16 (19.5)	138 (68.0)
					*p* < 0.001 ^+^
Teeth brushing frequency, n (%)					
Twice a day	344 (67.3)	179 (58.1)	131 (58.0)	48 (58.5)	165 (81.3)
Three or more times a day	167 (32.7)	129 (41.9)	95 (42.0)	34 (41.5)	38 (18.7)
					*p* < 0.001 ^+^
Sensodyne usage pattern, n (%) ^a^					
I used it every day	NA	223 (72.4)	223 (72.4)	NA	NA
I used it 4–5 days a week	NA	3 (1.0)	3 (1.0)	NA	NA
I used it 2–3 days a week	NA	25 (8.1)	NA	25 (30.5)	NA
I used it once every week	NA	2 (0.6)	NA	2 (2.4)	NA
I only used it for short periods of time until my dentine hypersensitivity/sensitive teeth improved, after which I switched to a different toothpaste	NA	55 (17.9)	NA	55 (67.1)	NA
I have used it together with another toothpaste (e.g., Pepsodent, Colgate, etc.) on the same day	NA	3 (1.0)	NA	3 (3.7)	NA
Last dentist review, n (%)					
Within past year	350 (68.5)	254 (82.5)	208 (92.0)	46 (56.1)	96 (47.3)
More than 1 year ago	128 (25.0)	48 (15.6)	17 (7.5)	31 (37.8)	80 (39.4)
Never	33 (6.5)	6 (1.9)	1 (0.4)	5 (6.1)	27 (13.3)
					*p* < 0.001 ^+^
Dentine hypersensitivity treatment, n (%) ^b^					
Oral medication (e.g., painkillers)	103 (20.2)	33 (10.7)	22 (9.7)	11 (13.4)	70 (34.5) ^+^
					*p* < 0.01 ^+^
Mouthwash	195 (38.2)	125 (40.6)	93 (41.2)	32 (39.0)	70 (34.5)
					*p* = 0.16 ^+^
Toothpaste for sensitivity	390 (76.3)	301 (97.7)	222 (98.2)	79 (96.3)	89 (43.8)
					*p* < 0.01 ^+^
Visited the dentist for consultation	184 (36.0)	149 (48.4)	131 (58.0)	18 (22.0)	35 (17.2)
					*p* < 0.01 ^+^
Other	6 (1.2)	0 (0.0)	0 (0.0)	0 (0.0)	6 (3.0)
I did not use any treatment for my dentine hypersensitivity/sensitive teeth	40 (7.8)	2 (0.6)	1 (0.4)	1 (1.2)	38 (18.7)
					*p* < 0.01 ^+^

Abbreviation: DH, dentine hypersensitivity; E-HT, episodic hypersensitivity toothpaste; HT, hypersensitivity toothpaste; NA, not applicable; R-HT, regular hypersensitivity toothpaste. * HT refers to Sensodyne Deep Clean/Sensodyne 24/7 Protection Fresh Mint/Sensodyne Cool Gel/Sensodyne Repair & Protect/Sensodyne Herbal/Sensodyne Original/Sensodyne Multi-Action. ^#^ MT refers to Pepsodent Pasta Gigi Berlubang/Pepsodent Complete 8 Herbal/Pepsodent Complete 8 Multi-Protection/Ciptadent Maxi Complete/Ciptadent Maxi Herbal/Close Up Everfresh. ^a^ This item was only completed by respondents who used HT. ^b^ As multiple options can be selected, the sum of proportions across all categories may exceed 100%. ^+^ Comparing MT users with HT users by paired/overlap Z-test for pecentages.

**Table 8:** Reasons for choosing HT (Sensodyne).

	HT * Users (N = 308)	R-HT * Users (N = 226)	E-HT * Users (N = 82)
Reason for choosing Sensodyne, n (%) ^a^			
Because it was recommended by my dentist/dental hygienist	102 (33.1)	86 (38.1)	16 (19.5)
Because I saw a TV or digital commercial of the product and decided to try it	157 (51.0)	108 (47.8)	49 (59.8)
Because my friends/family recommended it to me	149 (48.4)	118 (52.5)	31 (37.8)
Because I saw many positive reviews online (e.g., social media, blogs, social influencers, etc.)	79 (25.6)	64 (28.3)	15 (18.3)
Because it is good value for money	138 (44.8)	114 (50.4)	24 (29.3)
Because it often has promotions	25 (8.1)	21 (9.3)	4 (4.9)
Because I saw it displayed on the pharmacy/supermarket shelf and I decided to try it	113 (36.7)	83 (36.7)	30 (36.6)
Because a regular toothpaste is not enough to address my dentine hypersensitivity/sensitive teeth	210 (68.2)	157 (69.5)	53 (64.6)
Because I understand how Sensodyne works for sensitive teeth	155 (50.3)	113 (50.0)	42 (51.2)
Other reasons	2 (0.6)	1 (0.4)	1 (1.2)
Reason for using Sensodyne regularly, n (%) ^a,b^			
It was recommended by my dentist	NA	75 (33.2)	NA
I feel less dentine hypersensitivity after I use Sensodyne regularly	NA	195 (86.3)	NA
My teeth feels cleaner after using Sensodyne regularly	NA	152 (67.3)	NA
My breath smells fresh after using Sensodyne regularly	NA	119 (52.7)	NA
Besides clean teeth, my gums feel healthier after I use Sensodyne regularly	NA	156 (69.0)	NA
My teeth feels stronger after using Sensodyne regularly	NA	150 (66.4)	NA
Because I noticed that using Sensodyne regularly protects my teeth from cavities	NA	130 (57.5)	NA

Abbreviation: DH, dentine hypersensitivity; E-HT, episodic hypersensitivity toothpaste; HT, hypersensitivity toothpaste; NA, not applicable; R-HT, regular hypersensitivity toothpaste. * HT refers to Sensodyne Deep Clean/Sensodyne 24/7 Protection Fresh Mint/Sensodyne Cool Gel/Sensodyne Repair & Protect/Sensodyne Herbal/Sensodyne Original/Sensodyne Multi-Action. ^a^ As multiple options can be selected, the sum of proportions across all categories may exceed 100%. ^b^ This item was completed only by R-HT users.

**Table 9:** Attitudes and perception towards Sensodyne.

	HT * Users (N = 308)	R-HT * Users (N = 226)	E-HT * Users. (N = 82)
I enjoy Sensodyne’s flavor, n (%)			
Completely disagree	0 (0.0)	0 (0.0)	0 (0.0)
Somewhat disagree	4 (1.3)	1 (0.4)	3 (3.7)
Neither agree nor disagree	8 (2.6)	3 (1.3)	5 (6.1)
Somewhat agree	87 (28.2)	54 (23.9)	33 (40.2)
Completely agree	209 (67.9)	168 (74.3)	41 (50.0)
I can use Sensodyne every day, n (%)			
Completely disagree	0 (0.0)	0 (0.0)	0 (0.0)
Somewhat disagree	6 (1.9)	0 (0.0)	6 (7.3)
Neither agree nor disagree	12 (3.9)	1 (0.4)	11 (13.4)
Somewhat agree	65 (21.1)	33 (14.6)	32 (39.0)
Completely agree	225 (73.1)	192 (85.0)	33 (40.2)
With regular use of Sensodyne/with Sensodyne, I believe my teeth sensitivity won’t come back, n (%)			
Completely disagree	0 (0.0)	0 (0.0)	0 (0.0)
Somewhat disagree	2 (0.6)	1 (0.4)	1 (1.2)
Neither agree nor disagree	17 (5.5)	5 (2.2)	12 (14.6)
Somewhat agree	123 (39.9)	86 (38.1)	37 (45.1)
Completely agree	166 (53.9)	134 (59.3)	32 (39.0)
I believe that regular use of Sensodyne/Sensodyne is the best choice for my sensitive teeth, n (%)			
Completely disagree	0 (0.0)	0 (0.0)	0 (0.0)
Somewhat disagree	0 (0.0)	0 (0.0)	0 (0.0)
Neither agree nor disagree	8 (2.6)	3(1.3)	5 (6.1)
Somewhat agree	63 (20.5)	41 (18.1)	22 (26.8)
Completely agree	237 (76.9)	182 (80.5)	55 (67.1)
With regular use of Sensodyne/with Sensodyne, I believe it prevents my teeth sensitivity from getting worse, n (%)			
Completely disagree	2 (0.6)	2 (0.9)	0 (0.0)
Somewhat disagree	1 (0.3)	1 (0.4)	0 (0.0)
Neither agree nor disagree	9 (2.9)	5 (2.2)	4 (4.9)
Somewhat agree	73 (23.7)	44 (19.5)	29 (35.4)
Completely agree	223 (72.4)	174 (77.0)	49 (59.8)
How would you rate the product (Sensodyne)’s value for money in relation to your oral health? n (%)			
1—Poor value	1 (0.3)	0 (0.0)	1 (1.2)
2	0 (0.0)	0 (0.0)	0 (0.0)
3	11 (3.6)	7 (3.1)	4 (4.9)
4	81 (26.3)	51 (22.6)	30 (36.6)
5—Excellent value	215 (69.8)	168 (74.3)	47 (57.3)
How much do you trust the product to help manage your dentine hypersensitivity/sensitive teeth? n (%)			
Do not trust it at all	0 (0.0)	0 (0.0)	0 (0.0)
Somewhat do not trust it	0 (0.0)	0 (0.0)	0 (0.0)
Neither	3 (1.0)	1 (0.4)	2 (2.4)
Somewhat trust it	40 (13.0)	20 (8.8)	20 (24.4)
Completely trust it	265 (86.0)	205 (90.7)	60 (73.2)
How would you rate your overall satisfaction with your experience of using Sensodyne? n (%)			
Absolutely dissatisfied	0 (0.0)	0 (0.0)	0 (0.0)
Somewhat dissatisfied	0 (0.0)	0 (0.0)	0 (0.0)
Neither satisfied nor dissatisfied	1 (0.3)	0 (0.0)	1 (1.2)
Somewhat satisfied	47 (15.3)	22 (9.7)	25 (30.5)
Completely satisfied	260 (84.4)	204 (90.3)	56 (68.3)
Are you planning to purchase Sensodyne again? n (%)			
I would definitely not buy it again	6 (1.9)	3 (1.3)	3 (3.7)
I would probably not but it again	0 (0.0)	0 (0.0)	0 (0.0)
I am not sure whether I would buy it again or not	0 (0.0)	0 (0.0)	0 (0.0)
I would probably buy it again	37 (12.0)	15 (6.6)	22 (26.8)
I would definitely buy it again	265 (86.0)	208 (92.0)	57 (69.5)
How likely would you be to recommend Sensodyne to other consumers who are experiencing dentine hypersensitivity/sensitive teeth? n (%)			
I would definitely not recommend it to others	5 (1.6)	4 (1.8)	1 (1.2)
I would probably not recommend it to others	1 (0.3)	0 (0.0)	1 (1.2)
I am not sure whether I would recommend it or not to others	9 (2.9)	1 (0.4)	8 (9.8)
I would probably recommend it to others	79 (25.6)	41 (18.1)	38 (46.3)
I would definitely recommend it to others	214 (69.5)	180 (79.6)	34 (41.5)
How likely would you be to recommend Sensodyne to friends and family? n (%)			
I would definitely not recommend it	1 (0.3)	0 (0.0)	1 (1.2)
I would probably not recommend it	0 (0.0)	0 (0.0)	0 (0.0)
I am not sure whether I would recommend it or not	2 (0.6)	1 (0.4)	1 (1.2)
I would probably recommend it	57 (18.5)	31 (13.7)	26 (31.7)
I would definitely recommend it	248 (80.5)	194 (85.8)	54 (65.9)

Abbreviation: E-HT, episodic hypersensitivity toothpaste; HT, hypersensitivity toothpaste; R-HT, regular hypersensitivity toothpaste. * HT refers to Sensodyne Deep Clean/Sensodyne 24/7 Protection Fresh Mint/Sensodyne Cool Gel/Sensodyne Repair & Protect/Sensodyne Herbal/Sensodyne Original/Sensodyne Multi-Action.

**Table 10:** Mean (SD) score of attitudes and perceptions towards Sensodyne.

	HT * Users (N = 308)	R-HT * Users (N = 226)	E-HT * Users (N = 82)
Overall satisfaction	4.84 (0.38)	4.90 (0.30)	4.67 (0.50)
			*p* < 0.01 ^#^
Value for money	4.65 (0.58)	4.71 (0.52)	4.49 (0.71)
			*p* < 0.01 ^#^
Trust	4.85 (0.38)	4.90 (0.31)	4.71 (0.51)
			*p* < 0.01 ^#^
Continue to purchase it	4.80 (0.63)	4.88 (0.52)	4.59 (0.83)
			*p* < 0.01 ^#^
Likelihood to recommend it to other consumers	4.61 (0.72)	4.74 (0.64)	4.26 (0.78)
			*p* < 0.01 ^#^
Likelihood to recommend it to friends and family	4.79 (0.47)	4.85 (0.37)	4.61 (0.64)
			*p* < 0.01 ^#^

Abbreviation: E-HT, episodic hypersensitivity toothpaste; HT, hypersensitivity toothpaste; R-HT, regular hypersensitivity toothpaste. * HT refers to Sensodyne Deep Clean/Sensodyne 24/7 Protection Fresh Mint/Sensodyne Cool Gel/Sensodyne Repair & Protect/Sensodyne Herbal/Sensodyne Original/Sensodyne Multi-Action. ^#^ Comparing E-HT users with R-HT users by *t*-test for means.

## Data Availability

The data presented in this study are available on request from the corresponding author. The data are not publicly available due to ethical restrictions.

## Supplementary Materials

The following supporting information can be downloaded at: www.mdpi.com/article/10.3390/ijerph22020175/s1, S1: Full Online Questionnaire.

## Abbreviations

The following abbreviations are used in this manuscript:

DH	Dentine Hypersensitivity
DHEQ	Dentine Hypersensitivity Experience Questionnaire
E-HT	Episodic Hypersensitivity Toothpaste
HT	Hypersensitivity Toothpaste
MT	Mainstream Toothpaste
OHrQoL	Oral Health Quality of Life
QoL	Quality of Life
R-HT	Regular Hypersensitivity Toothpaste
SD	Standard Deviation
