# Supplementary material for: A Real-World Study on the Quality of Life of Consumers with Dentine Hypersensitivity and the Benefits of Hypersensitivity Toothpaste Use
Source: Int J Environ Res Public Health. 2025 Jan 27;22(2):175. doi: 10.3390/ijerph22020175 (PMC11855150; doi:10.3390/ijerph22020175)
Supplement: Supplementary file 1 [file ijerph-22-00175-s001.zip › ijerph-3435195-supplementary.pdf]

Title: A Real-World Study on the Quality of Life of Consumers with Dentine Hypersensitivity and The Benefits of Hypersensitivity Toothpaste Use

Supplementary File S1: Full online questionnaire

Let us first ask a few questions to see if the study is relevant to you. Please remember throughout the survey, there are no right or wrong answers.

S1. GENDER

Please choose your gender below:

Male
Female

S2. AGE

May we know your full age in years? ____________

Below 18	**TERMINATE**
18 – 25 years old	
26 – 50 years old	
51 – 65 years old	
Above 65	**TERMINATE**

S3. MONTHLY HOUSEHOLD INCOME

What is the total monthly income of your household before taxes or deductions?

Please choose one range that best describes your income.

Less than IDR 1,500,000	
IDR 1,500,000 to 2,700,000	
IDR 2,700,001 to 3,000,000	
IDR 3,000,001 to 4,500,000	
IDR 4,500,001 to 6,000,000	
IDR 6,000,001 and above	
Rather not say	**TERMINATE**

S4. AREAS LIVED IN

Which of the following regions do you currently live in?

Please select one answer.

Jakarta	
Sumatera	

Kalimantan	
Sulawesi	
Bali Nusra	
Central Java	
West Java	
East Java	
Other, please specify	**TERMINATE**
I do not live in Indonesia	**TERMINATE**

S5. SYMPTOMS P3M

Have you experienced any of the following conditions or symptoms in the **past 3 months**?

Please select as applicable.

Stomachache	
Sore throat	
Tooth sensitivity/sensitive teeth	**CONTINUE ONLY IF SELECTED**
Atopic dermatitis/eczema	
Skin rashes	
Allergy	
I did not suffer from any of the above in the past 3 months	**TERMINATE**

S6. DH DIAGNOSIS

Have you ever been diagnosed with sensitive teeth by a dentist?

Yes
No

S7. FOOD/DRINK SENSITIVITY

Do any of these types of food or drinks cause your teeth to hurt/ experience sensitivity in the **past 3 months**?

Hot	
Cold	
Sweet	
Sour	
None of these	**TERMINATE IF S6=No AND S7=None of these**

S8. PRODUCTS USED IN P3M

What types of personal hygiene products have you been using in the **past 3 months?**

Please select as applicable.

Lotion	
Shampoo	
Bodywash	

Toothpaste	**TERMINATE IF <u>NOT</u> SELECTED**
Mouthwash	
Hair conditioner	
None of the above	**TERMINATE**

S9. BRUSHING TEETH HABIT – FREQUENCY (TIME)

How often do you brush your teeth with toothpaste?

Less often than once a day	**TERMINATE**
Once a day	
Twice a day	
Three or more times a day	

S10. BRANDS USED P3M

In the **<u>past 3 months</u>**, which brand(s) of toothpaste have you used?

Please select as applicable .

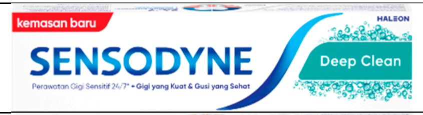	Sensodyne Deep Clean	
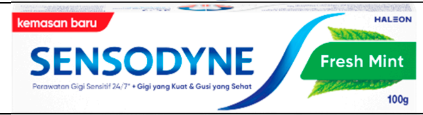	Sensodyne 24/7 Protection Fresh Mint	
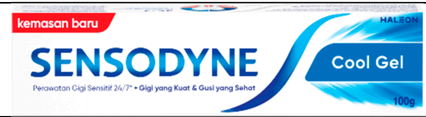	Sensodyne Cool Gel	
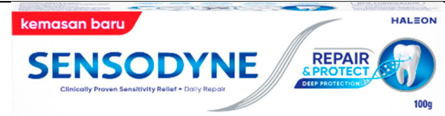	Sensodyne Repair & Protect	
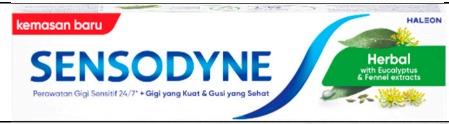	Sensodyne Herbal	
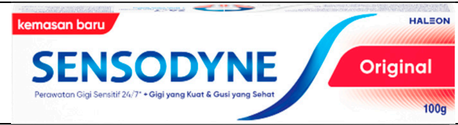	Sensodyne Original	
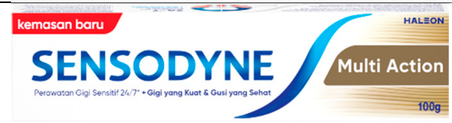	Sensodyne Multi-Action	
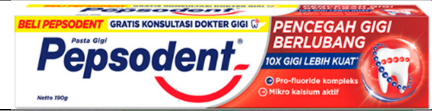	Pepsodent Pasta Gigi Berlubang	
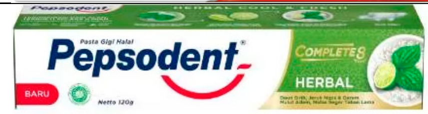	Pepsodent Complete 8 Herbal	

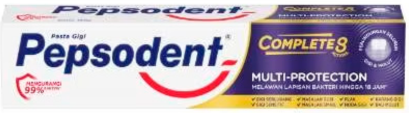	Pepsodent Complete 8 Multi-Protection	
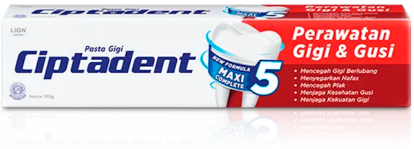	Ciptadent Maxi Complete	
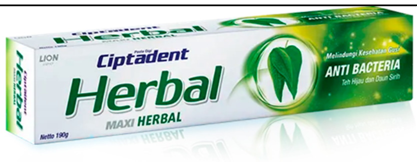	Ciptadent Maxi Herbal	
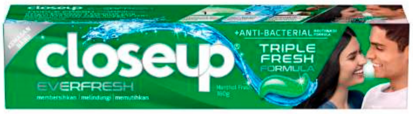	Close Up Everfresh	
Not available	Others. (Please specify: _____)	**TERMINATE IF NO RESPONDENT ONLY SELECTED 'Not available'**

S11. SENSODYNE USERS – USAGE PATTERN

Which of the following statements best describes how you used Sensodyne in the **past 3 months**?

I used it everyday
I used it 4-5 days a week
I used it 2-3 days a week
I used it once every week
I only used it for short periods of time until my dentine hypersensitivity/sensitive teeth improved, after which I switched to a different toothpaste
I have used it together with another toothpaste (e.g. Pepsodent, Colgate, etc) on the same day

S11B. SENSODYNE USERS – LAST USE

When was the last time you used Sensodyne?

Please select one answer.

One week ago or less
Two to three weeks ago
4 weeks / 1 month ago
2 months ago
3 months ago

S12. DENTAL PROFILE

Which of the following best describes your dental profile in the past 3 months?

Please select as applicable.

All of my teeth are my own natural teeth
I have most of my natural teeth and a few dental crowns/ bridges/ implants
I use fixed or removable orthodontic braces/ bands/ fixed orthodontic retainer
I use partial dentures
I use full dentures
I've had severe periodontal disease diagnosed by a dentist
I've had active tooth decay that was causing pain
I've had major dental surgeries (e.g. dental bone graft, periodontal surgery or jaw surgery)

S13. CHRONIC DISEASE

Do you have any chronic conditions which require you to **take pain medication for long term** in the past 3 months (E.g., you take pain medication more than 3 days a week)?

Yes	**TERMINATE**
No	

S14. AFFILIATION

Are you currently an employee or contractor to any of the companies listed below?

Market research company	**TERMINATE**
Advertising/PR company	
Pharmaceutical company	
Clinical research organization (CRO)	
Company manufacturing, distributing or selling consumer health prodcuts/personal care goods (e.g. dental hygiene products, skincare, haircare, etc.)	
Automotive	
Banking & Finance	
None of the above	

MAIN QUESTIONNAIRE

INTRODUCTION

Thank you for answering our questions so far.
The remainder of this questionnaire will focus on your experience of dentine hypersensitivity/sensitive teeth and it will take about 20 minutes to complete. Please take your time and answer the questions as accurately as possible. We would like to remind you that there are no right or wrong answers, and we only want to know your opinion. We very much appreciate your participation.

Q0. LAST DENTIST REVIEW

When was the last time you visited a dentist?

Within the last 6 months
6 – 12 months ago
1 – 5 years ago
More than 5 years ago
Never

Q0A. FREQUENCY OF DENTINE HYPERSENSITIVITY

You mentioned that you have experienced dentine hypersensitivity/ sensitive teeth in the past 3 months. How frequent did you experience dentine hypersentivity/ sensitive teeth?

Several times a day
Once a day
Several times a week
Once a week
Several times a month
Once a month
Less than once a month

Q0B. LASTING TIME OF DENTINE HYPERSENSITIVITY

And how long do you feel the dentine hypersensitivity/ sensitive teeth episode last for?

Less than a minute

1 – 10 minutes
11 – 20 minutes
21 – 30 minutes
More than 30 minutes

Q1. DENTINE HYPERSENSITIVITY/SENSITIVE TEETH MEDICATION

You mentioned that you have experienced dentine hypersensitivity/sensitive teeth in the past 3 months. Have you used any of the treatments below for it?

Oral medication e.g. painkillers
Mouthwash
Toothpaste for sensitivity
Visited the Dentist for consultation
Other
I did not use any treatment for my dentin hypersensitivity/sensitive teeth

ERROR! REFERENCE SOURCE NOT FOUND.. **DENTINE HYPERSENSITIVITY EXPERIENCE QUESTIONNAIRE (DHEQ) – FUNCTIONAL**

Tinking about yourself over the **last month**, to what extent would you agree or disagree with the following statements?

Please tick only one response for each question.

		Strongly agree	Agree	Agree a little	Neither agree nor disagree	Disagree a little	Disagree	Strongly disagree
1	Having the sensations in my teeth takes a lot of the pleasure out of eating and drinking							
2	It takes a long time to finish some foods and drinks because of these sensations in my teeth							
3	There have been times when I have had problems eating ice cream because of these							

	sensations							
4	I have to change the way I eat or drink certain things							
5	I have to be careful how I breathe on a cold day							
6	When eating some foods I have made sure they don't touch certain teeth							
7	Because of the sensations I take longer than others to finish a meal							
8	I have to be careful what I eat when I am with others because of the sensations in my teeth							
9	Going to the dentist is hard for me because I know it is going to be painful as a result of sensations in my teeth							
10	I've been anxious that something I eat or drink might cause sensations in my teeth							
11	The sensations in my teeth have been irritating							
12	The sensations in my teeth have been annoying							
13	Having these sensations in my teeth makes me feel old							
14	Having these sensations in my teeth makes me feel damaged							
15	Having these sensations in my teeth makes me feel as though I am unhealthy							

Q3. IMPACT LEVEL OF DH ON QUALITY OF LIFE

Thinking about your experience of dentin hypersensitivity/sensitive teeth, how much would you agree or disagree with each of the following statements?

Please select one response per statement.

		Completely disagree	Somewhat disagree	Neither agree nor disagree	Somewhat agree	Completely agree
1	I am uncomfortable to laugh freely when I am experiencing dentin hypersensitivity/sensitive teeth					
2	Dentin hypersensitivity/sensitive teeth restricts me from enjoying myself in social events					
3	Dentin hypersensitivity/sensitive teeth makes it hard for me to fall asleep					
4	Dentin hypersensitivity/sensitive teeth makes					
5	I cannot enjoy my morning coffee as much when I have dentin hypersensitivity/sensitive teeth					
6	I have been restricting myself from sugary snacks and drinks to avoid dentin hypersensitivity/sensitive teeth episodes					
7	I have to reduce my alcohol consumption due to dentin hypersensitivity/sensitive teeth					

Q3A. BRAND BEFORE SENSODYNE

You mentioned that you have used Sensodyne in the past 3 months. Have you ever tried or used a different brand of toothpaste before using Sensodyne?

Yes
No

Q4. REASON OF CHOOSING SENSODYNE – EPISODIC AND REGULAR USERS

You mentioned that you have used Sensodyne in the past 3 months. Could you please share why you **[FOR Q3A=1, SHOW** "switched to"**, FOR Q3A=2, SHOW** "chose"**]** Sensodyne?

Please select as applicable.

Because it was recommended by my dentist/ dental hygienist
Because I saw a TV or digital commercial of the product and decided to try it
Because my friends/family recommended it to me

Because I saw many positive reviews online (e.g. social media, blogs, social influencers, etc.)
Because it is good value for money
Because it often has promotion
Because I saw it displayed on the pharmacy / supermarket shelf and I decided to try it
Because a regular toothpaste is not enough to address my dentine hypersensitivity/sensitive teeth
Because I understand how Sensodyne works for sensitive teeth
Other reason

Q5. REASON FOR REGULAR USE

Can you tell us the reason(s) to why you use Sensodyne regularly?

It was recommended by my dentist
I feel less dentine hypersensitivity after I use Sensodyne regularly
My teeth feels cleaner after using Sensodyne regularly
My breath smells fresh after using Sensodyne regularly
Besides clean teeth, my gums feel healthier after I use Sensodyne regularly
My teeth feels stronger after using Sensodyne regularly
Because I noticed that using Sensodyne regularly protects my teeth from cavities
Other reason

Q6. SENSODYNE CLAIMS – ALL SSD USERS

Thinking about your experience in using Sensodyne, how much would you agree or disagree with each of the following statements?

Please choose one response per statement.

		Completely disagree	Somewhat disagree	Neither agree nor disagree	Somewhat agree	Completely agree
1	Since using Sensodyne, I am able to eat the food that I like without worrying about tooth sensitivity					

2	I enjoy Sensodyne's flavour					
3	I can use Sensodyne everyday					
4	My dental hypersensitivity has improved after using Sensodyne					
5	I experience less frequent dental hypersensitivity episodes after using Sensodyne					
6	Sensodyne provides better relief for my dental hypersensitivity as compared to other toothpaste brands I have used					

Q6A. SENSODYNE CLAIMS – REGULAR USERS

Thinking about your experience in using Sensodyne, how much would you agree or disagree with each of the following statements?

Please choose one response per statement.

		Completely disagree	Somewhat disagree	Neither agree nor disagree	Somewhat agree	Completely agree
1	With regular use of Sensodyne, I can enjoy eating sweets and desserts without being bothered by tooth sensitivity					
2	With regular use of Sensodyne, I believe my teeth sensitivity won't come back					
3	I believe that regular use of Sensodyne is the best choice for my sensitive teeth					
4	With regular use of Sensodyne, I believe it prevents my teeth sensitivity from getting worse					
5	Regular use of Sensodyne provides me long-lasting					

	feeling of fresh breath					
6	Regular use of Sensodyne cleans my teeth well					
7	With regular use of Sensodyne, I feel less pain when I have hot and cold food and drinks					

Q6B. SENSODYNE CLAIMS – EPISODIC USERS

Thinking about your experience in using Sensodyne, how much would you agree or disagree with each of the following statements?

Please choose on response per statement.

		Completely disagree	Somewhat disagree	Neither agree nor disagree	Somewhat agree	Completely agree
1	With Sensodyne, I can enjoy eating sweets and desserts without being bothered by tooth sensitivity					
2	With Sensodyne, I believe my teeth sensitivity won't come back					
3	I believe that Sensodyne is the best choice for my sensitive teeth					
4	With Sensodyne, I believe it prevents my teeth sensitivity from getting worse					
5	Sensodyne provides me long-lasting feeling of fresh breath					
6	Sensodyne cleans my teeth well					
7	With Sensodyne, I feel less pain when I have hot and cold food and drinks					

Q7. VALUE FOR MONEY

Thinking about your experience of using Sensodyne in the past 3 months, how would you rate the product's value for money in relation to your oral health?

Please give a rating on a scale from 1 to 5, where 1 means "Poor value for money", and 5 means "Excellent value for money".

**Poor value**	1	2	3	4	5	**Excellent value**

Q8. TRUST

Thinking about your experience of using Sensodyne in the past 3 months, how much do you trust the product to help manage your dentine hypersensitivity/sensitive teeth?

Please choose one response.

Do not trust it at all
Somewhat not trust it
Neither
Somewhat trust
Completely trust

Q9. OVERALL SATISFACTION

From your experience of using Sensodyne, how would you rate your overall satisfaction with it?

Please choose one response.

Absolutely dissatisfied
Somewhat dissatisfied
Neither satisfied nor dissatisfied
Somewhat satisfied
Completely satisfied

Q10. CONTINUED PURCHASE

And are you planning to purchase Sensodyne again?

Please choose one response below.

I would definitely not buy it again
I would probably not but it again

I am not sure whether I would buy it again or not
I would probably buy it again
I would definitely buy it again

Q11. LIKELIHOOD OF RECOMMENDATION (OTHERS)

How likely would you be to recommend Sensodyne to other consumers who are experiencing dentine hypersensitivity/sensitive teeth?

Please choose one response below that best describes your opinion.

I would definitely not recommend it to others
I would probably not recommend it to others
I am not sure whether I would recommend it or not to others
I would probably recommend it to others
I would definitely recommend it to others

Q12. LIKELIHOOD OF RECOMMENDATION (FAMILY & FRIENDS)

How likely would you be to recommend Sensodyne to friends and family?

Please choose one response below that best describes your opinion.

I would definitely not recommend it
I would probably not recommend it
I am not sure whether I would recommend it or not
I would probably recommend it
I would definitely recommend it

-Those were all the questions we have today. Thank you for your participation!
We greatly value your opinion.-
